# Circulatory microRNA 23a and microRNA 23b and polycystic ovary syndrome (PCOS): the effects of body mass index and sex hormones in an Eastern Han Chinese population

**DOI:** 10.1186/s13048-016-0298-8

**Published:** 2017-02-13

**Authors:** Weixi Xiong, Ying Lin, Lili Xu, Amin Tamadon, Shien Zou, Fubo Tian, Ruijin Shao, Xin Li, Yi Feng

**Affiliations:** 1 Department of Integrative Medicine and Neurobiology, State Key Lab of Medical Neurobiology, School of Basic Medical Sciences, Shanghai Medical College; Institute of Acupuncture Research (WHO collaborating center for traditional medicine) and Institute of Brain Science, Brain Science Collaborative Innovation Center, Fudan University, Shanghai, 200032 China; 2 Grade 2008 Clinical Medicine, Shanghai Medicine School, Fudan University, Shanghai, 200032 China; 3 Department of Neurology, West China Hospital of Sichuan University, Chengdu, Sichuan 610041 China; 4 Department of Medical Oncology, Fudan University Shanghai Cancer Center, Shanghai, 200032 China; 5 Department of Cardiology, Zhongshan Hospital, Fudan University, Shanghai, 200032 China; 6 Department of Gynecology, Obstetrics and Gynecology Hospital, Fudan University, Shanghai, 200011 China; 7 Institute of Neuroscience and Physiology, Department of Physiology, Sahlgrenska Academy, University of Gothenburg, Gothenburg, 40530 Sweden

**Keywords:** microRNAs, miR-23a/b, Obesity, Sex hormones, Polycystic ovary syndrome

## Abstract

**Background:**

MicroRNAs (miRNAs) regulate the expression of genes involved in various cellular functions related to metabolism, inflammation, and reproduction. This study evaluated the effects of sex hormones and obesity on the expression of circulating miR-23a and miR-23b in women with polycystic ovary syndrome (PCOS) and healthy women.

**Methods:**

Serum sex hormones concentrations and body mass index (BMI) were measured in 18 women with PCOS and in 30 healthy women from the East China area and these measurements were correlated with serum miR-23a/b levels. The effect of miR-23a and miR-23b risk factors on occurrence of PCOS and predisposing factors of PCOS on these miRNA expressions were evaluated.

**Results:**

The expressions of miR-23a/b were significantly lower in the women with PCOS than the normal women, and the expression levels of miR-23a/b were positively correlated with each other in the normal women (*p* = 0.001) but not in the women with PCOS (*p* > 0.05). In the women with PCOS, miR-23a was positively correlated with BMI (*p* = 0.03). However, no correlations were found between the levels of miR-23a/b and the sex hormones in the normal and PCOS women. On the other hand, without considering the presence or absence of PCOS, increase in BMI had a positive effect on the levels of circulating miR-23b; while testosterone had negative effects on the levels of circulating miR-23a. Furthermore, the likelihood of women with PCOS decreased by 0.01-fold for every 1 fold increase of miR-23a expression.

**Conclusions:**

Both reduced levels and discordance between the expressions of miR-23a/b were observed in the women with PCOS and miR-23a/b were affected from testosterone and BMI, reversely. Therefore, miR-23a alteration in contrast with miR-23b is a better indicator for evaluation of PCOS than the miR-23b.

## Background

Polycystic ovary syndrome (PCOS) is one of the most common reproductive, endocrine, and metabolic disorders in women. It affects about 5 to 10% of women of reproductive age and is usually a lifelong disease. PCOS is characterized by chronic anovulation, hyperandrogenism, and, consequently, infertility [1]. Metabolic disorders, including obesity, insulin resistance, and diabetes, are cofactors as well as predisposing factors of PCOS [2]. Therefore, understanding the molecular mechanisms of the metabolic diseases underlying the pathophysiology of PCOS will help to identify novel diagnostic and therapeutic strategies.

Previously, it was shown that microRNAs (miRNAs) play an important role in follicular development and fertility [3]. The miRNAs are highly conserved, 19 to 25 nucleotide-long, single-stranded RNA molecules that post-transcriptionally regulate gene expression, and they perform their functions by mediating translational repression or by directing the cleavage of target mRNAs. Recent evidence suggests that miRNAs play fundamental roles in all cellular and tissue activities under both normal and pathological conditions, but the currently available information on the expression and function of miRNAs in the ovary, especially in oocyte development, is still very limited. It was discovered, however, that miRNAs might be involved in the turnover of many maternal transcripts whose degradation might be essential for the successful completion of meiotic maturation by oocytes [3].

The analysis of expression of miR-23a and miR-23b in follicular cells from women undergoing assisted reproductive technology (ART) including the patients with tubal factor and endometriosis showed that significant increase in the levels of miR-23b directly correlated with CYP19A1 (aromatase gene) expression, miR-23a, compared to normal women [4]. Aromatase which convert androgens to estrogen has role in pathogenesis of PCOS [5]. Therefore, in the present study, serum samples and other information were collected from women with PCOS and healthy controls to study the correlation between serum miR-23a/b expression, obesity, and sex hormones in women with PCOS.

## Results

### Expression of miR-23a/b in controls and PCOS patients

**Fig. 1:**
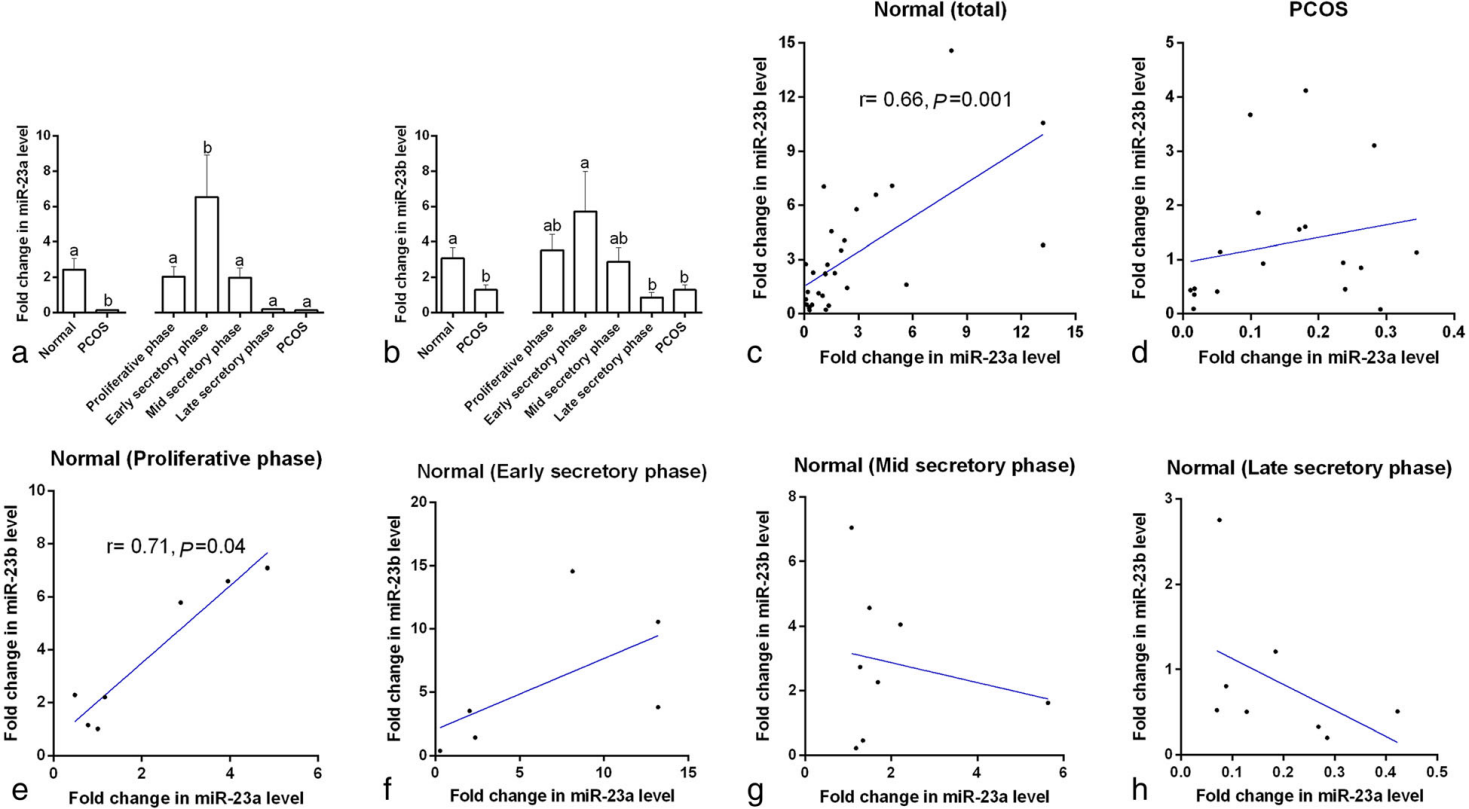
Serum miRNA-23a (**a**) and miRNA-23b (**b**) expression in normal women, polycystic ovary syndrome (PCOS) patients, and normal women at different stages of the menstrual cycle. ^a,b^ different *superscript letters* indicate significant differences between groups (*p* < 0.05). Correlation between serum miRNA-23a and miRNA-23b expression in normal women (**c**) and PCOS patients (**d**) and in normal women at different stages of the menstrual cycle (**e**-**h**). The scatter plots with “r” and “*p*” values given are the ones with significant correlation coefficients (*p* < 0.05)

The expression of miR-23a and miR-23b was down-regulated in the women with PCOS compared to the healthy controls (C_v_ = 73.2, *p* = 0.008, and C_v_ = 93.3, *p* = 0.04, respectively, Fig. 1a and b). Comparison of the healthy women according to the different phases of the endometrial cycle (proliferative, early secretory, mid-secretory, and late secretory) showed that miR-23a and miR-23b reached peak concentrations in the early secretory phase and were at their lowest level in the late secretory phase (*p* < 0.05, Fig. 1a and b). Furthermore, there was a positive correlation between the expression of miR-23a and miR-23b in the serum of the healthy controls and in the subgroup of controls in the proliferative phase (C_v_ = 142.4 and C_v_ = 108.9, respectively, *p* < 0.05, Fig. 1c and e), but there was no significant correlation between the expression of the two miRNAs in the women with PCOS (*r* = 0.3) or in healthy controls in the mid-secretory (*r* = −0.1), early secretory (*r* = 0.7), or late secretory (*r* = −0.6) phases.

### Body mass index (BMI) and expression of miR-23a/b in PCOS patients and healthy controls

**Fig. 2:**
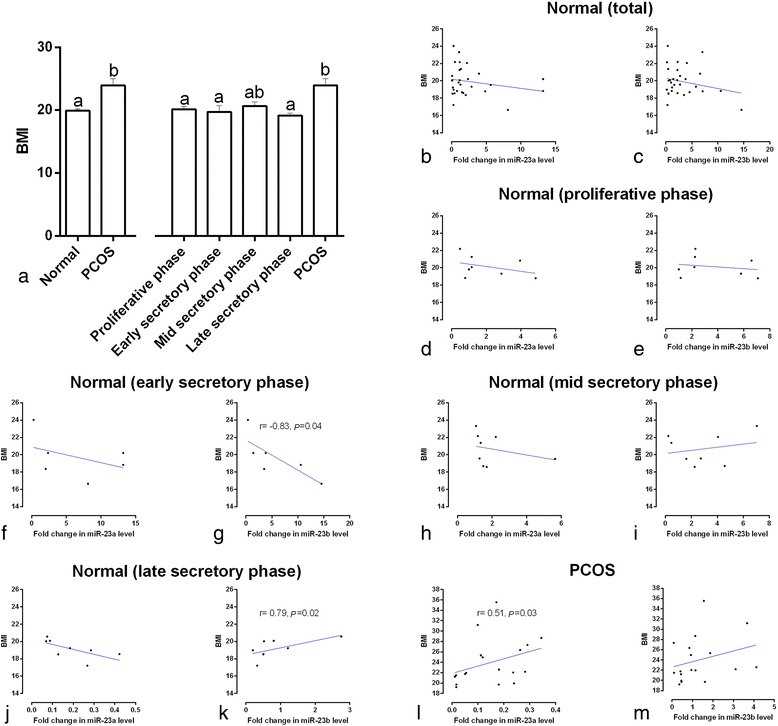
Body mass index (BMI, kg/m^2^) (**a**) in normal women, polycystic ovary syndrome (PCOS) patients, and normal women at different stages of the menstrual cycle. ^a,b^ different *superscript letters* indicate significant differences between groups (*p* < 0.05). Correlation of serum miRNA-23a and miRNA-23b expression with BMI in normal women (**a** and **b**), normal women at different stages of the menstrual cycle (**c**–**k**), and PCOS patients (**l** and **m**). The scatter plots with “r” and “*p*” values given are the ones that had significant correlation coefficients (*p* < 0.05)

The BMI in the women with PCOS was higher than in the healthy controls as a group (C_v_ = 18.5 vs. C_v_ = 8.4, respectively; *p* = 0.001) and in the proliferative, early secretory, and late secretory subgroups (*p* < 0.05, Fig. 2a). There was a positive correlation between BMI and the expression of miR-23a in the serum of PCOS patients (*p* < 0.05, Fig. 2l), but no such correlation was seen in any of the healthy controls (*p* > 0.05). On the other hand, BMI and serum miR-23b expression showed significant negative and positive correlations in the early and late secretory phases in normal women, respectively, (*p* < 0.05, Fig. 2g and k), but not in the proliferative or mid-secretory subgroups or in the PCOS patients (*r* = 0.4, *p* = 0.09, Fig. 2m).

### Testosterone and expression of miR-23a/b in PCOS patients and healthy controls

**Fig. 3:**
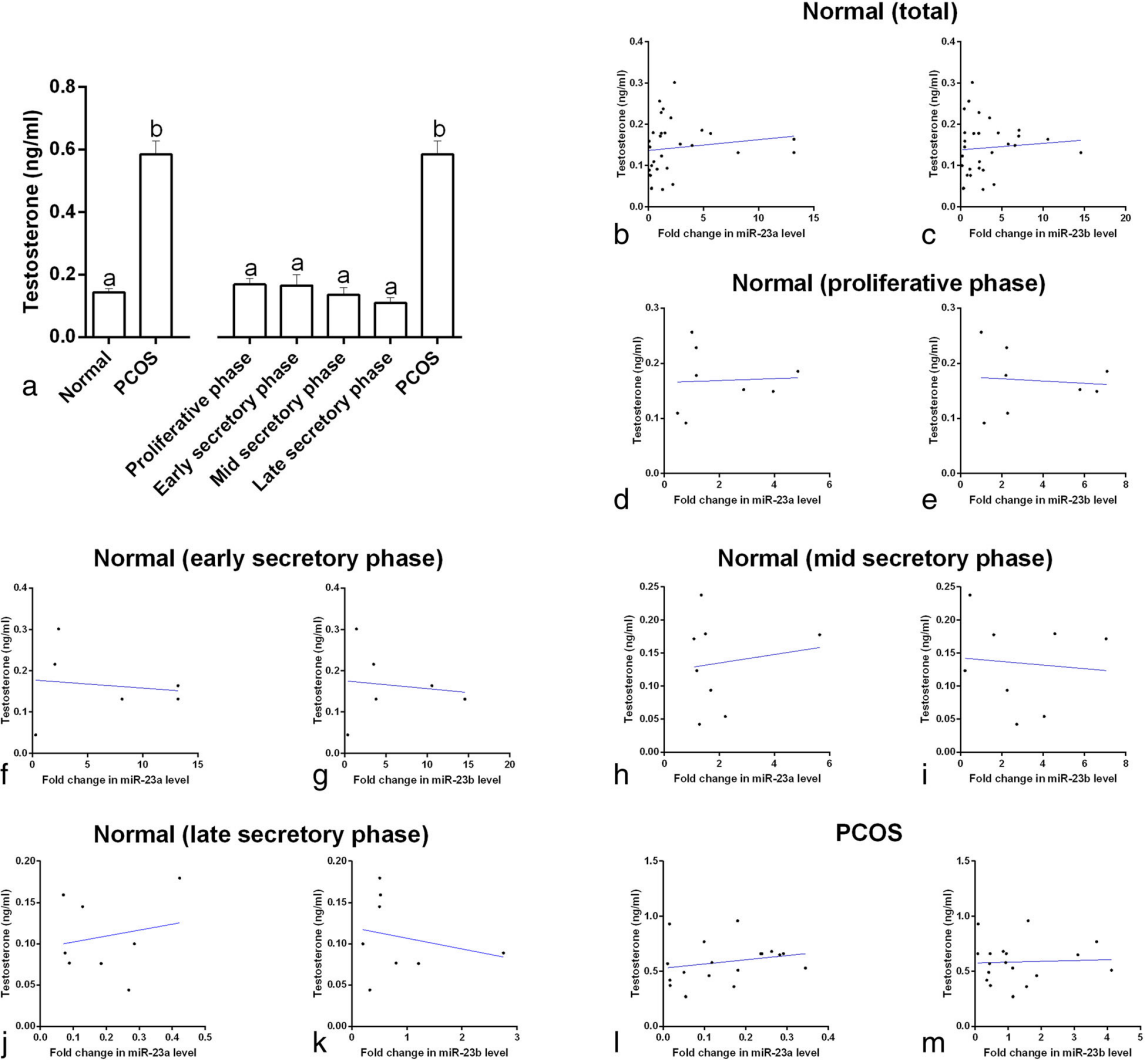
Serum testosterone concentrations (**a**) in normal women, polycystic ovary syndrome (PCOS) patients, and normal women at different stages of the menstrual cycle. ^a,b^ different *superscript letters* indicate significant differences between groups (*p* < 0.05). Correlation of serum miRNA-23a and miRNA-23b expression with testosterone concentrations in normal women (**a** and **b**), in normal women at different stages of the menstrual cycle (**c**–**k**), and in PCOS patients (**l** and **m**). None of the scatter plots showed significant correlation coefficients (*p* > 0.05)

The serum testosterone concentrations in the PCOS patients were about four times higher than in the healthy controls (C_v_ = 31.6 vs. C_v_ = 46.2, respectively) and in the four subgroups of healthy controls (*p* < 0.001, Fig. 3a). However, there was no correlation between miR-23a or miR-23b expression and testosterone concentrations in the serum of the PCOS patients or the healthy controls in any phase of the endometrial cycle (*p* > 0.05, Fig. 3b to m).

### P_4_ and E_2_ and expression of miR-23a/b in healthy controls

**Fig. 4:**
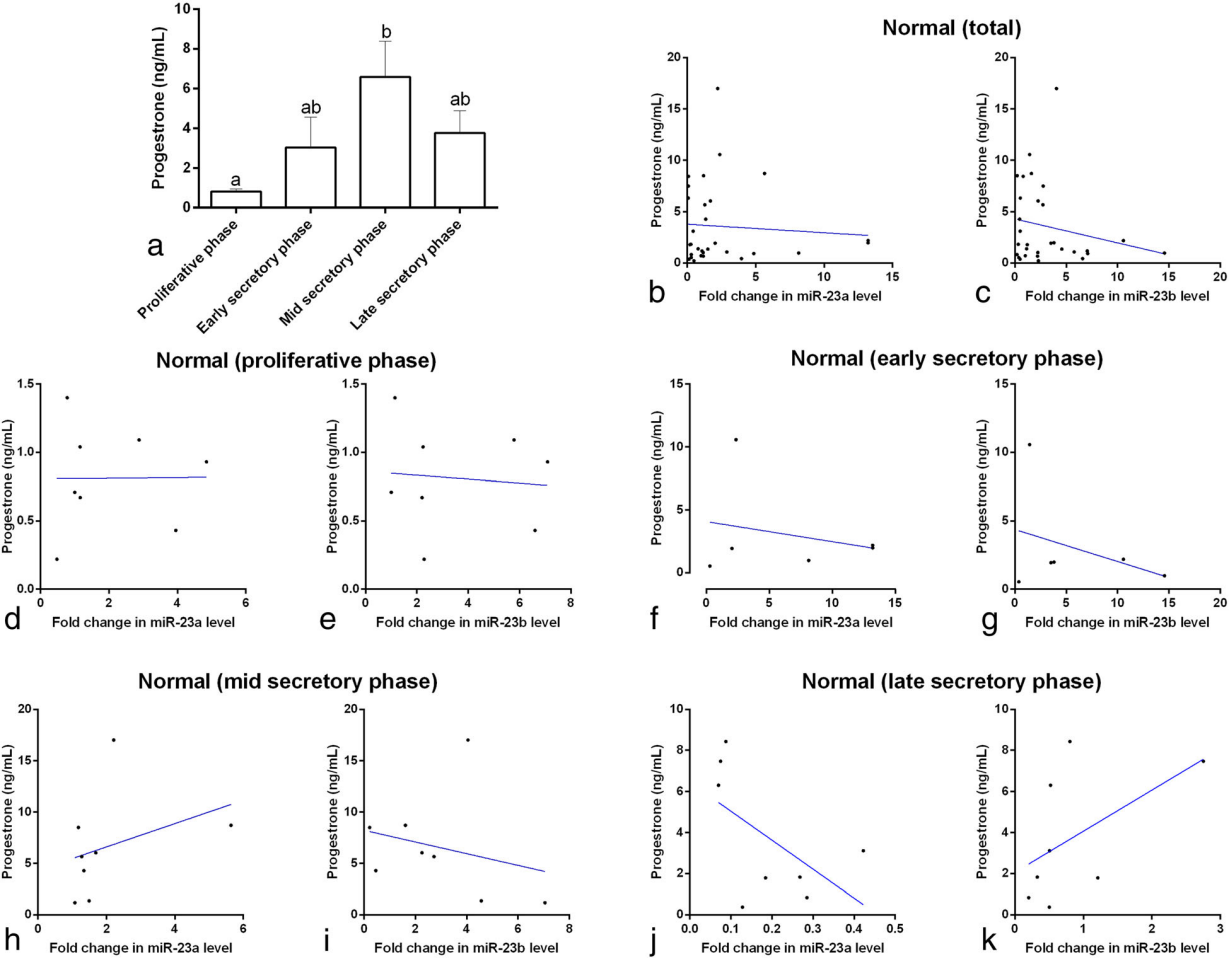
Serum progesterone concentrations (**a**) in normal women at different stages of the menstrual cycle. ^a,b^ different *superscript letters* indicate significant differences between groups (*p* < 0.05). Correlation of serum miRNA-23a and miRNA-23b expression with progesterone in normal women (**a** and **b**), in normal women at different stages of the menstrual cycle (**c**–**k**), and in PCOS patients (**l** and **m**). The scatter plots showed no significant correlation coefficients (*p* > 0.05)

**Fig. 5:**
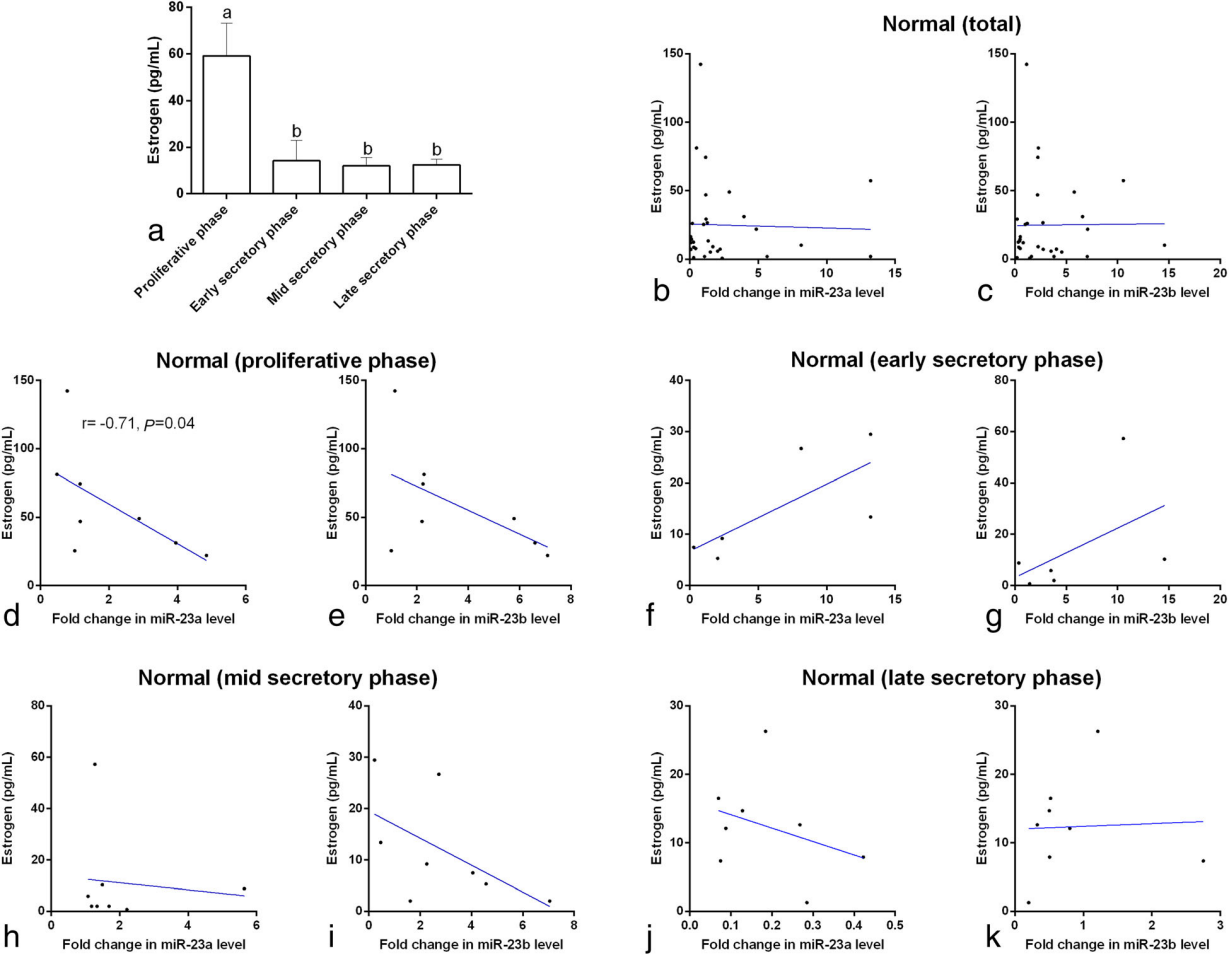
Serum estrogen concentrations (**a**) in normal women at different stages of the menstrual cycle. ^a,b^ different *superscript letters* indicate significant differences between groups (*p* < 0.05). Correlation of serum miRNA-23a and miRNA-23b expression with estrogen in normal women (**a** and **b**), in normal women at different stages of the menstrual cycle (**c**–**k**), and in PCOS patients (**l** and **m**). The scatter plots showed no significant correlation coefficients (*p* < 0.05)

Similarly to what has been demonstrated in previous studies, the serum P_4_ and E_2_ concentrations reached their peak levels in the mid-secretory phase and proliferative phase, respectively (*p* < 0.05, Figs. 4a and 5a), and were at their the lowest concentrations in the proliferative phase and mid-secretory phase, respectively (*p* < 0.05). In the healthy controls in the proliferative phase, the E_2_ concentrations were negatively correlated with the expression of miR-23a (*p* = 0.04, Fig. 5d), but not with the expression of miR-23b (*p* > 0.05, Fig. 5e). There was no correlation between P_4_ concentration and miR-23a or miR-23b expression (*p* > 0.05) in the proliferative phase nor were there any correlations between E_2_ and P_4_ and miR-23a or miR-23b expression in any of the other phases.

### Luteinizing hormone (LH) and follicle stimulating hormone (FSH) and expression of miR-23a/b in PCOS patients

**Fig. 6:**
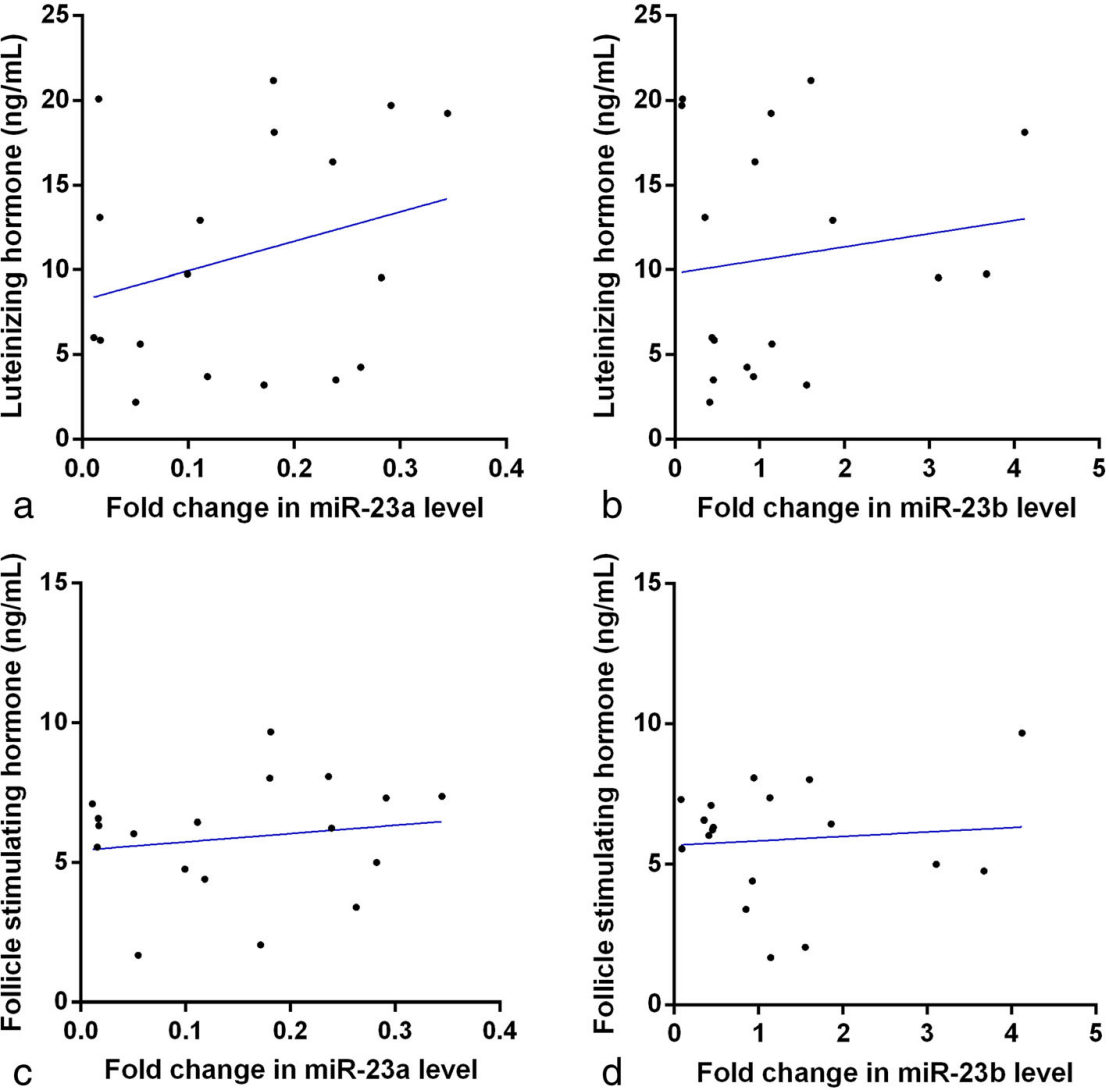
Correlation of serum miRNA-23a and miRNA-23b expression with luteinizing hormone (LH, **a** and **b**) and follicle stimulating hormone (FSH, **c** and **d**) concentrations in PCOS patients. The scatter plots showed no significant correlation coefficients (*p* > 0.05)

The mean and SE values of LH and FSH concentrations in PCOS patients were 10.8 ± 1.6 ng/mL and 5.9 ± 0.5 ng/mL, respectively, and did not significantly correlate with miR-23a or miR-23b expression (Fig. 6).

### Hormones and BMI in healthy controls and PCOS patients

Concentrations of testosterone (*r* = 0.04), LH (*r* = −0.06), and FSH (*r* = −0.3) did not correlate with BMI in the PCOS patients (*p* > 0.05). The correlation coefficients between testosterone concentrations and BMI were negative in all four subgroups of healthy women, but none of the correlations were statistically significant (*p* > 0.05). Among the healthy controls, the correlation coefficients between E_2_ and P_4_ concentrations and BMI were not significant, except for the correlation between P_4_ concentrations and BMI in the late secretory phase (*r* = 0.71, *p* = 0.04).

### Power of study and odds ratio of effective factors on PCOS and expression of miR-23a/b

The observed powers of analysis in the risk factors (miR-23a/b expression, BMI and testosterone concentrations) of PCOS were more than 80% (Table 1). Furthermore, the adjusted odds ratios of the variables finally included in the logistic model are shown in Tables 2 and 3. Logistic regression analysis indicated significant effects of the fold changes of miR-23a/b on the likelihood of women with PCOS. The likelihood of women with PCOS decreased by 0.01-fold for every 1 fold increase of miR-23a expression (*P* = 0.02; Table 2). On the other hand, the miR-23b expression on the PCOS was not significant in the equation (*P* > 0.05; Table 2).

**Table 1:** Observed powers of analysis regarding to different dependent variables in five groups of normal and polycystic ovary syndrome women

Variables	Observed power (%)
miRNA-23a	100
miRNA-23b	93
Body mass index	95
Testosterone	100

**Table 2:** Odds ratios of the variables included in the final logistic regression model for the polycystic ovary syndrome and serum miRNA-23a and miRNA-23b expression

Variables	Odds ratio	95% Confidence interval	*P*-value
miRNA-23a	0.012	0.0–0.46	0.017
miRNA-23b	1.803	0.99–3.54	0.053

Backward likelihood ratio test = 28.57, 2 df, *P* = 0.0001; Hosmer and Lemeshow goodness-of-fit test = 2.81, 8 df, *P* = 0.95; the model fits

**Table 3:** Odds ratios of the variables included in the final logistic regression model for the serum miRNA-23a or miRNA-23b expression and body mass index and testosterone concentration

Variables	Odds ratio	95% Confidence interval	*P*-value
miRNA-23a			
Body mass index	1.051	0.99–1.11	0.08
Testosterone	0.003	0.00–0.24	0.01
miRNA-23b			
Body mass index	1.056	1.01–1.11	0.03
Testosterone	0.100	0.01–1.47	0.09

miRNA-23a: Backward likelihood ratio test = 14.08, 2 df, *P* = 0.001; Hosmer and Lemeshow goodness-of-fit test = 18.67, 8 df, *P* = 0.02; the model fits

miRNA-23b: Backward likelihood ratio test = 5.17, 2 df, *P* = 0.08; Hosmer and Lemeshow goodness-of-fit test = 8.47, 8 df, *P* = 0.39; the model fits

Without considering the presence or absence of PCOS, logistic regression analysis indicated significant negative effects of the testosterone concentrations on the likelihood of fold changes of miR-23a (Table 3). The likelihood of more than 1 in fold changes of miR-23a would be 0.003-fold for each 1 fold decrease in testosterone concentration (*P* = 0.01). While, BMI significant positive effect was observed only on the likelihood of fold changes of miR-23b. The likelihood of more than 1 in fold changes of miR-23b would be 1.056-fold for each 1 fold increase in BMI (*P* = 0.03).

### Biological functions of the predicted targets of miR-23a/b

**Fig. 7:**
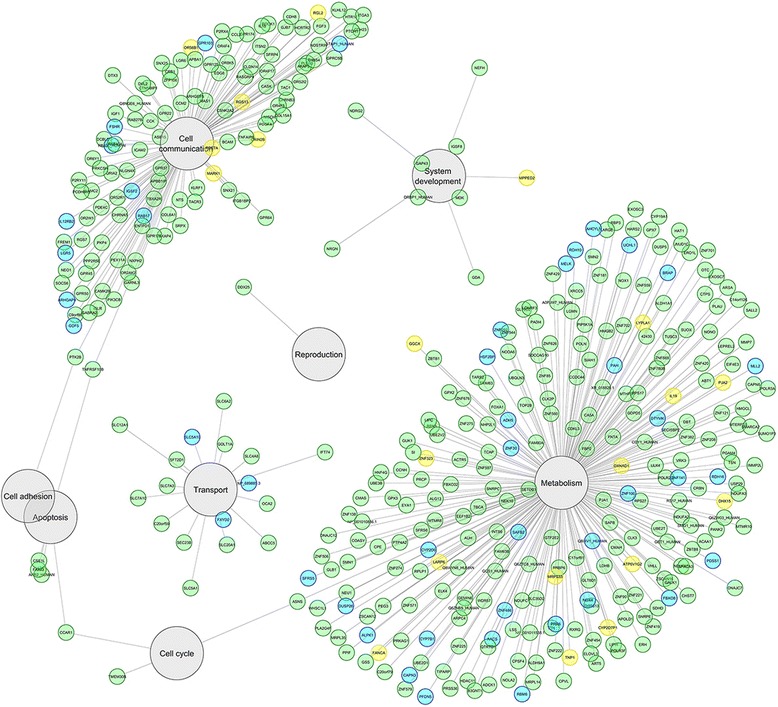
Mapping of target genes that were common to miRNA-23a (*blue nodes*), miRNA-23b (*yellow nodes*), or both miRNAs (*green nodes*) to biological processes (*grey nodes*). The landscape of genes was mapped to biological processes and visualized using the Cytoscape 3.4.0 software [21]. The lengths of the edges show the relative scores for the effects of the miRNAs on the target genes according to the MicroCosm Targets software

The predicted target genes for miR-23a and miR-23b were screened in *Homo sapiens* using the MicroCosm Targets software. The list of predicted target genes included 1078 genes for miR-23a and 1049 genes for miRNA-23b, from which 377 and 356 target genes, respectively, were mapped to biological functions and processes, including hormone synthesis, metabolic functions, and sexual reproduction. Of these target genes, the analysis revealed 309 common target genes for both miR-23a and miR-23b, 68 genes related exclusively to miR-23a, and 47 genes related exclusively to miR-23b, and these genes are involved in eight biological processes (Fig. 7).

## Discussion

We found a negative influence of decrease of miR-23a on occurrence of PCOS and increase of testosterone. On the other hand, although a positive effect of BMI on the expression levels of miR-23b (without considering presence or absence of PCOS) was observed in logistic regression analysis, but decrease in miR-23b fold-changes as well as miR-23a decrease was observed in PCOS women. Considering this fact that miR-23a alterations was not affected by BMI in contrast with miR-23b, suggests it as a better indicator for evaluation of PCOS than the miR-23b.

Furthermore, the BMI among the women with PCOS was higher on average compared with the healthy controls. The mean BMI of healthy women of the same ethnicity and in the same age range (20.99 ± 3.31 kg/m^2^) [6] is similar to the BMI among healthy controls in the current study. Therefore, all of the PCOS patients in this study could be considered to be obese (the mean BMI of the PCOS patients was 23.96 ± 4.44 kg/m^2^). Obesity in the PCOS patients increased the expression of both miR-23a and miR23b, but BMI was correlated with decreased expression of these miRNAs in the healthy controls. The pattern of correlations between obesity and the expression of miR-23a and miR23b that was observed in the present study resembles the pattern of correlations of serum testosterone, LH, and FSH concentrations with the expression of the miR-23a and miR23b, but the effects of obesity on the relationship with miR-23a/b expression in the women with PCOS was greater than the association of hormone changes with miR-23a/b expression. In the healthy controls and the endometrial-phase subgroups, obesity and testosterone concentrations had negative correlation coefficients, whereas in the women with PCOS a positive but non-significant correlation in increased BMI and testosterone concentrations was observed. Consistent with our findings, Murri et al. [7] reported opposite patterns of association between obesity and testosterone concentrations in PCOS patients and healthy controls. According to the normal range of testosterone levels among healthy women of the same ethnicity and in the same age range of the current study (0.32 ± 0.16 ng/mL) [6], all of the PCOS patients who were selected in this study based on the Rotterdam consensus criteria were defined as having hyperandrogenemia (0.59 ± 0.19 ng/mL).

The patterns of changes in the expression of miR-23a and miR-23b were the same in the PCOS patients and in the healthy controls and in the endometrial-phase subgroups, and in the present study the expressions of both miR-23a and miR-23b in serum were significantly lower in the women with PCOS compared to the healthy controls. The one exception was that the mean expression of miR-23b in the women with PCOS was higher than that in the healthy controls in the late secretory phase, and it can be speculated that this difference might be due to the involvement of miR-23b in ovulation. In support of this, it has been shown that miR-23b targets the X-linked inhibitor of apoptosis and can induce apoptosis in human granulosa cells in vitro [8]. In addition, a comparison of seasonally ovulatory and anovulatory follicles in horses revealed increased expression of miR-23b in the anovulatory follicles [9]. Furthermore, in our current work we found that the pattern of expression of miR-23a and miR-23b changed from a positive correlation in the proliferative phase to a negative correlation in the late secretory phase; whereas the women with PCOS showed a positive correlation in the expressions of miR-23a and miR-23b.

In the present study, the increase in serum E_2_ concentrations in the proliferative phase was negatively correlated with the expression of miR-23a and miR-23b in the healthy controls. Furthermore, in the evaluated population, a negative influence of increase of testosterone concentrations on miR-23a expression was observed. In addition, the same non-significant effect of testosterone was observed on miR-23 expression in whole blood. A previous study on the expression of miR-23a and miR-23b in follicular fluid showed that expression of these miRNAs along with the expression of their target gene could regulate the expression of aromatase, CYP19A1, in ovarian cells and, therefore, might have a role in E_2_ biosynthesis [4]. Therefore, it can be speculated that alterations in the expression of these miRNAs in serum might affect follicular growth and ovulation via other target genes than those that play a role in E_2_ hormone synthesis, including target genes that are functionally related to cell growth and apoptosis. In the present study, a decrease in miR-23a expression in women with PCOS was observed, and overexpression of pre-miR-23 has previously been shown to play a role in apoptosis in cultured human luteinized granulosa cells [8]. Therefore, altered miR-23a expression in PCOS patients might induce down-regulation of apoptotic processes in ovarian cells.

With the help of bioinformatics tools, we have shown that miR-23a and miR-23b target large numbers of genes and that many of these genes are targeted by several other miRNAs. Target genes of miR-23a and miR-23b are involved in many biological functions, including metabolic, cellular, and reproductive processes that are important in PCOS pathogenesis [2, 10]. One of the metabolic disorders that has a definite role in PCOS is obesity [11], and several studies have shown that cellular communication can be altered in PCOS and obesity, including communication between inflammatory cells and metabolic cells [12–14]. Additionally, inflammatory and immune gene targets that regulate cellular processes such as apoptosis can influence follicular function and steroid production [10, 15].

The major limitation of the present study was possibly the evaluation of serum miRNA expression, which represents miRNAs from several unknown origin cell types. However, several studies have highlighted the potential of serum samples to act as developmental markers of diseases, including PCOS [7, 16–18]. Therefore, sampling of serum might represent the overall state of the entire body instead of specific cells at the time of collection.

## Conclusions

The present research showed lower concentrations of miR-23a and miR-23b in the serum of PCOS patients compared to healthy controls. Furthermore, we demonstrated the positive influence of obesity on the serum expression of miR-23b related to metabolic and cell function disorders. In addition, testosterone had negative effects on the levels of circulating miR-23a. Exploring the target genes and pathways of miR-23a/b and other differentially expressed miRNAs will contribute to a better understanding of the roles of miRNAs in the pathogenesis of PCOS. In terms of new biomarkers for the detection of PCOS in patients, miR-23a might be a better choice, but the correlation between the levels of these miRNAs in the serum and in the follicular cells needs further investigation.

## Methods

### Subjects and selection criteria

In the current study, 18 Han Chinese women (with a mean ± SD age of 25.8 ± 4.5 years) were recruited from the Affiliated Obstetrical and Gynecological Hospital of Fudan University between September 2011 and January 2012. The women were all diagnosed with PCOS based on the revised diagnostic criteria announced in the Rotterdam consensus [1], and patients with Cushing syndrome, late-onset congenital adrenal hyperplasia, thyroid dysfunction, hyperprolactinemia, or androgen-secreting tumors were excluded. Other exclusion criteria included diabetes, hypertension, chronic renal disease, smoking, and the use of alcohol or medications. Thirty healthy age-matched Han Chinese women (25.5 ± 2.3 years old) with no previous history of reproductive system diseases or appendicitis served as controls. The control women had normal and regularly cycling menstrual periods, and their ovaries appeared normal on ultrasound. The exclusion criteria of the healthy women in the study were taking drugs, including oral contraceptives or other hormone drugs, intrauterine device placement, smoking, and/or pregnancy in the past 3 months. Control subjects were divided into four groups according to their endometrial cycle phase – proliferative phase (days 4–14, *n* = 8), early secretory phase (days 15–18, *n* = 6), mid-secretory phase (days 19–24, *n* = 8), and late secretory phase (days 25–30, *n* = 8).

### Assessment of BMI and sex hormones

The BMI in both normal women and PCOS patients was calculated as weight (kg) divided by the square of the height (m^2^). Measurements and blood samples were conducted within the first 10 days from the onset of menstruation in PCOS cases with mild oligomenorrhea, and they were conducted at random times for PCOS cases with severe oligomenorrhea or amenorrhea. Measurements and blood samples were conducted at different phases of the endometrial cycle in controls as described above. Total testosterone, LH, and FSH were measured by radioimmunoassay (RigorBio Scientific and Technology Co., Beijing) according to the manufacturer’s instructions.

### Quantification of miR 23a/b in peripheral blood

Venous blood samples (5 ml) were drawn from every subject. Serum was separated by centrifuging at 3000 × *g* for 10 min at 4 °C and was stored at −20 °C. Whole RNA was extracted from 200 μL of serum with the miRcute miRNA Isolation kit (DP501, Tiangen Biotech, Beijing) according to the manufacturer’s instructions. The RNA was then reverse transcribed using the miRcute miRNA first-strand cDNA synthesis kit (KR201, Tiangen Biotech, Beijing), and quantitative real-time polymerase chain reaction (qPCR) was performed using the miRcute miRNA qPCR detection kit (Tiangen Biotech, Beijing). The qPCR was performed under the following conditions: initial PCR denaturation at 94 °C for 120 s followed by 42 combined cycles of denaturation of 20 s at 94 °C and annealing and extension of 34 s at 60 °C. Fluorescence was measured at 55 °C in 81 cycles of 10 s. Results were calculated using the 2^−ΔΔCt^ method, and U6 was used as the controls for miR-23a and miR-23b. The sequences of primers were as follows (Invitrogen, Shanghai):

Primer sequence of miR-23a: 5′-ATCACATTGCCAGGGATTTCCA-3′

Primer sequence of miR-23b: 5′-GCACATTGCCAGGGATTACCA-3′

U6 as the internal control: 5′-CTCGCTTGGGCAGCACA-3

### Statistical analysis

Data are described as the mean ± SD. An independent sample *t*-test or one-way ANOVA with correction of *p*-values with the Bonferroni *post-hoc* test was used to test for differences in demographic variables and laboratory measurements between PCOS patients and healthy controls. Spearman correlation coefficients were calculated to evaluate the relationship between miRNA levels and other measurements in both the PCOS and control groups. All data were analyzed using SPSS version 22.0 (SPSS, Inc., Chicago, IL), and *p* < 0.05 was considered statistically significant.

To evaluate the power of study regarding to the selected sample size and the five groups of normal and PCOS women, the observed power of the dependent variables (miR-23a/b expression, BMI and testosterone concentrations) were estimated using univariate analysis of variance in general linear model of SPSS [19].

Possible effects of the miR-23a and miR-23b on occurrence of PCOS were explored using logistic regression analysis. The data of the normal women was used as reference. The data were compared by logistic regression analysis using the presence of PCOS as the dependent variable (0 denotes normal and 1 denotes PCOS) and the expression of miR-23a and the expression of miR-23b as the independent factors were entered into equation. Furthermore, possible effects of the BMI and serum testosterone concentration on the fold change of miR-23a and miR-23b were explored using logistic regression analysis. The data of the fold change of miR-23a/b less than 1 was used as reference. The data were compared by logistic regression analysis using the fold change of miR-23a or miR-23b as the dependent variable (0 denotes less than 1 fold change and 1 denotes more than or equal 1 fold change) and the BMI and serum testosterone, concentration as the independent factors were entered into equation.

Regression analyses were conducted according to the method of Hosmer and Lemeshow [20]. The *p*-values for data inclusion and exclusion were set at 0.05 and 0.10, respectively. The variable that had been selected or retained entered the final likelihood ratio (LR), in which the final odds ratio estimates with 95% confidence intervals were derived. The constants were not included in the model.

## Abbreviations

BMI: Body mass index
FSH: Follicle stimulating hormone
LH: Luteinizing hormone
miRNA: microRNA
PCOS: Polycystic ovary syndrome
qPCR: Quantitative real-time polymerase chain reaction
